# The embodied experience of abstract art: Moving across the 20th century

**DOI:** 10.1177/03010066251329918

**Published:** 2025-04-03

**Authors:** Ralf F. A. Cox, Lisa-Maria van Klaveren

**Affiliations:** University of Groningen, the Netherlands; University of Groningen, the Netherlands

**Keywords:** art experience, embodiment, postural control, recurrence quantification analysis, emotion, aesthetic appraisal, abstract art, paintings

## Abstract

The aim of this study was to contribute to our understanding of embodied art experiences. We were interested in the emerging relationship between artwork, on the one hand, and one's bodily movements and associated embodied affective states on the other. Concretely, postural control of 46 participants looking at a diverse set of 21 20th-century abstract paintings was analysed. Also, we explored the relation between postural control, emotional states of being moved and aesthetic appraisal. Results did not reveal differences in postural control between the paintings. However, differences in variability, dynamic stability, complexity and intermittency of postural sway were found, when comparing subsets of high-motion and low-motion paintings and between subclasses of abstract painting styles. Emotional states of being moved and aesthetic appraisal were associated with postural control, both across paintings and across people in several ways. Together these findings provide empirical evidence for an embodied art experience.

## Introduction

Art is renowned for its capacity to move people physically and emotionally. Following pragmatic and phenomenological traditions, the experience of art is an embodied activity (Dewey, 1934; Merleau-Ponty, 2010). The embodied activity is reflected in the intricate link between the artwork's spatial and temporal characteristics, such as motion or perspectival features, and the spectator's bodily movements (e.g., Van Geert & Wagemans, 2020). To explain this link, Freedberg and Gallese (2007) hypothesize that aesthetic experiences involve mirror neurons and embodied simulation. That is, neural mechanisms enable a spectator to simulate the actions and emotions of the subjects depicted in the artwork and/or of the artist during the creation of the work. For instance, bodily involvement in art experiences can be observed in (artistic) movement simulation (Leder et al., 2012). Their study demonstrated that aesthetic pleasure is enhanced when the movements made by participants (stroking or stippling) while viewing the painting match those belonging to the artistic style (stroking or pointillist) of the painter during art creation.

This account has been challenged by Brinck (2018), who points out that it might not be able to incorporate the reciprocity aspect of empathy through art and fails to explain what makes some experiences aesthetic. Instead, actual bodily movements go hand in hand with emotional states of being moved and with aesthetic appraisals such as beauty (Brinck, 2018; Kühnapfel et al., 2024; see also Fuchs & Koch, 2014). Research in this area provides a glimpse of the embodied, dynamic and integrative nature of art experiences (Brinck, 2018). It emphasizes that the regularities observed in motion, emotion and cognition during art experiences emerge from the interactions between the many processes going on in and between a spectator and an artwork, following principles of self-organisation (Cox et al., 2023).

### Postural Control in Art Experiences

Several studies have shown how posture is influenced by cognitive performance and, conversely, how body posture affects cognition and attention. For instance, a decrease in the Stroop effect has been measured between participants who were standing compared to those who were sitting (Rosenbaum et al., 2017). Similarly, Bertamini et al. (2013) showed that the perceived attractiveness of female bodies was higher when the posture of the participant and of the displayed body (i.e., sitting versus standing) were compatible.

Postural sway refers to the subtle, continuous, and involuntary movements around the body's centre of gravity while standing, which not only play an important role in maintaining our balance but also in the way we interact with our environment. It has been shown that attentional demands impact postural sway (e.g., Pellecchia, 2003), and that the nature of this impact depends on the type of cognitive task that is performed concurrently (e.g., Ramenzoni et al., 2007). Furthermore, postural control is affected by the processing of visual stimuli, and this, in turn, is influenced by people's emotional state (Lelard et al., 2019). The exact nature of the relation between cognition and postural control involves many interacting factors (for an overview see Salihu et al., 2022; also see Ganczarek et al., 2024).

Postural control has been used as a window on embodied art experiences. For instance, Kapoula et al. (2011, 2015; Kapoula & Gaertner, 2015) propose that pictorial depth and (illusory) motion present in figurative paintings lead to more postural sway. This could be directly caused by such “visual cues,” but also (changes in) fixations and mental imagery have also been proposed to play a role (Ganczarek et al., 2015). Recently, Ganczarek et al. (2024) studied people's postural control in relation to semantic information about paintings being watched. They showed how cognitively challenging art including conceptual ambiguities influenced postural-sway variability and postural dynamics in various ways. In a recent study (Cox & Van Klaveren, 2024), we showed how postural dynamics was different between looking at neoplastic paintings by Piet Mondriaan and looking at action paintings by Jackson Pollock. These results on art-elicited postural control solicited the question as to their generalizability, at least to other abstract painting styles. To put it boldly, to what extent is postural control characteristic of a specific abstract painting or abstract painting style? It remains to be seen whether other types of expressive spatial configurations used by abstract painters, which may vary in more subtle ways, also lead to detectable differences in the postural-control measures. Similarly, do the links between the subjective experience and postural control, as revealed in the previous study, generalize to other paintings and styles?

### The Present Study

Interestingly, art's motion and perspectival features are continuously changing based on historical and individual movements with peak abstractions throughout the 20th century (Wölfflin, 2015; Heft, 2022). To investigate the questions above, the present study included a selection of abstract paintings spanning a broad range of artistic styles during this period. In addition, the present study has a dualistic focus with respect to the embodied-emotional link in the art experience, analysing both across-painting propensities and across-person predispositions. That is, here we also investigated whether individual participants’ overall postural-sway patterns and their overall embodied perceptions of movement and aesthetic appreciations are associated, in addition to analysing such associations with respect to the paintings.

Although postural sway clearly has the potential to provide valuable insights into the embodied nature of art experiences, and similarly in other subfields of perceptual science, it still is relatively unexploited as a research method. The present study has two aims: To showcase the use of postural sway and to add to the empirical record of embodied art experiences. Participants viewed a variety of abstract paintings spanning a broad range of artistic styles. In addition to their postural sway, we also measured participants’ feelings of being moved-by and drawn-towards the paintings as well as their aesthetic appraisal of the paintings in terms of beauty and quality. Guiding general research questions were: To what extent is postural dynamics reflective of a specific abstract painting (style)? Can we identify characteristics of painting styles that elicit different patterns of postural sway? How are postural dynamics related to emotional states of being moved and aesthetic appraisals, both across participants and across paintings?

## Method

### Participants

Data of 46 undergraduate students of the University of Groningen (aged 18–25 years; 38 females and 8 males) were successfully collected, in exchange for course credits. Data of 14 more participants were discarded from further analyses because of equipment malfunction or violation of the procedure. All participants had normal or corrected-to-normal vision, and none of them reported colour blindness. They were naïve to the aims of the study. Recruitment was done with flyers in university buildings and through the university's online research participants system SONA. Those interested contacted one of the researchers to make an appointment in the lab. The study was approved by the local ethics committee of the Department of Psychology at the University of Groningen.

### Materials and Procedure

Participants viewed 21 carefully selected images of abstract paintings, while standing without shoes on a Wii Balance Board (Nintendo Company, Ltd.). The Wii Balance Board is a validated instrument to register postural sway (e.g., Koslucher et al., 2012; Pavan et al., 2014; Clark et al., 2018). The images were displayed on a large monitor (178 × 99 cm; 1,920 × 1,080 pixels; 60 Hz; Samsung Group), for 45 s at a distance of 1.9 m. For an overview of all the paintings see Supplement 1. All selected paintings were created in the 20th century and reflected different styles reaching from constructivism to abstract expressionism. Limited by the dimensions of the monitor, the height of all stimuli was set to 90 cm (26.6° visual angle) while keeping the proportions of the original painting intact. The visual angles of the width of the paintings ranged from 30.3° to 36.2°. Paintings were presented in a random order, in three blocks of seven with a short break between each block using OpenSesame (version 3.3.). This was synchronized with a custom-built software application, communicating with the Wii Balance Board through a Bluetooth wireless connection, which registered postural-sway data at a fixed sample rate of 100 Hz. After its presentation, the painting disappeared from the screen and the postural sway measurement ended. Next, participants answered four items on a scale from 1 to 10 using a tablet (iPad; Apple, Inc.) running Qualtrics, in a self-paced manner, with the respective painting being visible again. The items assessed participants’ emotional state of being moved in terms of being “Moved-By” (“How much are you emotionally moved by the painting that you have just seen?”) and “Drawn-Towards” (“How much are you emotionally drawn towards the painting that you have just seen?”), as well as their aesthetic appreciation in terms of “Beauty” (“In your opinion, how beautiful is this painting?”) and Quality (“In your opinion, how much aesthetic merit/quality does the painting possess?”). After the tablet was retrieved from the participant, the next painting was presented. The entire experiment lasted about half an hour, including (optional) breaks and debriefing.

### Data Analysis

Postural control was analysed in terms of the centre-of-pressure (COP) movement in the medial-lateral (ML; side-to-side) direction and anterior-posterior (AP; back-and-forth) direction. Movement analyses were performed on 30 s of measurement (i.e., 3,000 data points), after low-pass Butterworth filtering. Several COP measures were considered. Leaning posture (i.e., the overall tendency of leaning left/right and towards/away) was operationalized as the average of the COP movements in each direction (AV_ML_ and AV_AP_). The spatial magnitude of the postural-sway variability was operationalized as the standard deviation of the COP movements in each direction (SD_ML_ and SD_AP_). To more fully exploit the subtle and rich information contained in COP movements, recurrence quantification analysis (RQA) was performed. This provided several measures reflecting the dynamic organization of postural control (DET, MNL, ENT, LAM, TT, in each direction). RQA is explained in Supplement 2 (also see Cox & Van Klaveren, 2024; Ganczarek et al., 2024). Determinism (DET_ML_ and DET_AP_) refers to the extent of deterministic patterns in the COP movements. Meanline (MNL_ML_ and MNL_AP_) is a measure of the dynamic stability of the postural dynamics. Entropy (ENT_ML_ and ENT_AP_) quantifies the dynamic complexity of the postural dynamics. Laminarity (LAM_ML_ and LAM_AP_) and Trapping Time (TT_ML_ and TT_AP_) capture the proportion and average duration of laminar states in the COP movements, respectively. Laminar states represent intermittency or rigidity in a system, that is, when the system gets stuck in one or more states for some period of time.

We used G*Power to do the power calculations. For the comparison between the three abstract painting styles, using α = 0.05, *f *= 0.25 (medium effect size), and a power of 0.8, the calculation yielded a minimum of 28 participants. For the comparison between the static versus dynamic paintings, using α = 0.05, *f *= 0.25 (medium effect size), and a power of 0.8, the calculation yielded a minimum of 34 participants. For the initial comparison between all 21 paintings, we expected a small effect size. Using α = 0.05, *f *= 0.1, and a power of 0.8, this calculation yielded a minimum of 51 participants. Finally, based on our previous work, we expected quite a strong correlation between the (averaged) ratings and (averaged) movement variables. Using *α* = 0.05, *ρ* = 0.5, and a power of 0.8, this calculation yielded a minimum of 26 participants.

Because of the non-normality of the distributions of the COP measures, the statistical analyses were performed using non-parametric tests. To detect differences between (groups of) paintings, Friedman and Wilcoxon tests with Monte-Carlo resampling (10,000 samples) were done. Spearman's rank correlations were calculated across paintings between the over-participants-averaged COP measures and ratings. First, averages over all participants were calculated for each painting. These averaged COP measures and ratings were subsequently correlated across paintings. This reveals the extent to which bodily movements are associated with emotional states and appreciation during art experiences, using the paintings (rather than people) as individual probes. Similarly, Spearman's rank correlations were calculated across participants between the over-paintings-averaged COP measures and ratings. Extreme outliers (≥|3 SD|) with respect to COP total path length were removed. No corrections for multiple comparisons were made in this exploratory study (Armstrong, 2014).

## Results

Table 1 shows the postural-control measures and ratings for the emotional state of being moved and aesthetic appraisal, averaged over all 21 paintings in the experiment with standard deviations. Friedman tests yielded significant differences between paintings for all questions: Moved-By, χ^2^(20) = 117.84, *p *< .001, *W *= .20, and Drawn-Towards, χ^2^(20) = 105.48, *p *< .001, *W *= .18, and for Beauty, χ^2^(20) = 104.77, *p *< .001, *W *= .18, and Quality, χ^2^(20) = 115.48, *p *< .001, *W *= .20. So, on average, some paintings were considered more beautiful and of higher aesthetic quality and caused participants to feel more moved by and drawn towards than others did. Contrary to our expectations, none of the COP measures significantly differed between paintings. This null result is in itself not very informative. Therefore, below we will present additional exploratory analyses on subsets of the total sample of paintings, in order to reveal characteristics of the paintings or painting styles related to postural dynamics.

**Table 1.** Averages and standard deviations of the COP measures in both movement directions and of the ratings for the four questions over all the paintings, as well as separated for the most static (“low-motion”) paintings and most dynamic (“high-motion”) paintings and for the three abstract painting styles (i.e., fragmentary, organic, and dynamic).

	Medio-lateral							Anterior-posterior							Questions			
	AV	SD	DET	MNL	ENT	LAM	TT	AV	SD	DET	MNL	ENT	LAM	TT	MVD	DRW	BTY	QTX
All paintings																		
Average	−0.76	0.32	0.67	50.3	3.84	0.77	48.6	2.22	0.53	0.80	63.1	4.09	0.88	56.4	4.00	4.15	5.37	5.85
St. Dev.	1.88	0.45	0.14	16.1	0.32	0.11	13.7	1.85	0.35	0.09	20.1	0.28	0.06	12.4	2.33	2.41	2.33	2.18
High-motion paintings																		
Average	−0.72	0.37	0.69	53.7	3.90	0.79	51.3	2.31	0.58	0.80	62.5	4.10	0.88	56.3	4.31	4.12	4.36	5.21
St. Dev.	1.30	0.45	0.12	18.0	0.28	0.09	15.8	1.75	0.39	0.08	15.4	0.23	0.06	9.59	2.25	2.36	1.98	2.07
Low-motion paintings																		
Average	−0.65	0.36	0.67	49.2	3.81	0.77	47.4	2.20	0.51	0.79	60.9	4.07	0.87	54.3	2.94	3.20	4.44	5.04
St. Dev.	1.45	0.47	0.12	10.8	0.24	0.09	9.72	1.93	0.33	0.08	14.9	0.22	0.05	8.87	1.83	2.00	2.11	2.02
Fragmentary-abstract paintings																		
Average	−0.77	0.29	0.68	50.2	3.85	0.78	48.6	2.33	0.53	0.80	63.7	4.10	0.88	56.4	3.79	4.07	5.43	6.02
St. Dev.	1.39	0.18	0.11	10.4	0.23	0.08	9.12	1.69	0.27	0.07	17.0	0.22	0.05	9.96	1.75	1.98	1.59	1.58
Organic-abstract paintings																		
Average	−0.79	0.29	0.67	50.8	3.85	0.77	48.4	2.19	0.51	0.80	63.3	4.10	0.88	56.7	4.17	4.52	5.66	6.25
St. Dev.	1.22	0.26	0.11	12.9	0.24	0.08	10.3	1.74	0.27	0.07	14.4	0.21	0.05	9.21	1.65	1.78	1.47	1.41
Dynamic-abstract paintings																		
Average	−0.72	0.38	0.69	52.7	3.89	0.78	50.9	2.31	0.56	0.80	63.8	4.11	0.88	57.0	4.33	4.35	4.91	5.56
St. Dev.	1.51	0.53	0.12	12.9	0.24	0.09	11.7	1.68	0.31	0.08	16.4	0.23	0.05	9.85	1.80	1.89	1.57	1.55

### Dynamic Versus Static Paintings

Here, we explored the distinction that has been made between “low-motion” and “high-motion” paintings (Humphries et al., 2021). We instructed five researchers (including the authors) to choose the three most dynamic (“high-motion”) paintings and the three most static (“low-motion”) paintings out of the 21 in the original sample. The four paintings that clearly stood out were selected for comparison, two being the top-rating dynamic ones (nrs. 11 and 20 in Supplement 1), the other two being the top-rating static ones (nrs. 3 and 7 in Supplement 1), where the (averaged) postural-sway measures were expected to be significantly lower for the latter motion category. For the average values of all the measures separated for the two motion categories, see Table 1. One-tailed Wilcoxon tests revealed differences for several of the COP measures: MNL_ML_, *Z *= –1.913, *p *= .027, *r *= .20, ENT_ML_, *Z *= –2.421, *p *= .007, *r *= .25, TT_ML_, *Z *= –1.800, *p *= .035, *r *= .19, SD_AP_, *Z *= –1.778, *p *= .037, *r *= .19, and, TT_AP_, *Z *= –1.755, *p *= .040, *r *= .18. This means that the high-motion paintings elicited more dynamically stable, complex and intermittent postural sway, which was also more variable in the back-and-forth direction, than the low-motion paintings did. Two-tailed Wilcoxon tests showed that participants rated the high-motion paintings significantly higher on Moved-By, *Z *= –3.314, *p *= .001, *r *= .43, and on Drawn-Towards, *Z *= –2.203, *p *= .030, *r *= .28.

### Comparing Abstract Painting Styles

The second exploratory analysis focused on whether different abstract painting styles elicited different postural-sway patterns in viewers. To this end, together with an art historian, we categorized the paintings in the original sample, based on their painting style, historical context, and artists’ intentions into three groups: fragmentary-abstract, organic-abstract, and dynamic-abstract. The stylistic and historical categorization was informed by Brettell (1999) and Britt (2013). Information from available catalogues, publications, and descriptions of museums (e.g., Tate Modern) were used to describe individual artworks, as can be found in Supplement 3. In total 13 paintings were grouped, as follows: nrs. 4, 9, 15, 19 for fragmentary-abstract, nrs. 1, 2, 13, 18 for organic abstract, and nrs. 5, 11, 12, 14, 20 for dynamic abstract (see Supplement 3). Table 1 shows the average values of all measures separated for the three abstract painting styles. We performed separate two-tailed Wilcoxon tests to check for differences between the three abstract painting style categories. We found less variable postural sway in both movement directions for organic-abstract style paintings compared to dynamic-abstract style paintings, *Z *= –2.399, *p *= .016, *r *= .25 (SD_ML_), and *Z *= –2.139, *p *= .032, *r *= .22 (SD_AP_). No differences in the COP measures were found between fragmentary-abstract style paintings and dynamic-abstract style paintings and between fragmentary-abstract and organic-abstract style paintings. Finally, dynamic-abstract paintings were rated as less beautiful and of lower quality than organic-abstract paintings, *Z *= –2.862, *p *= .004, *r *= .30, and *Z *= –3.370, *p *= .001, *r *= .35, respectively.

### Across-Painting Propensities of Art Experience

There were associations between the measures of the emotional states of being moved and aesthetic appraisals on the one hand and the COP measures on the other hand (both averaged over all participants for each of the 21 paintings separately). Beauty ratings of paintings are significantly correlated with the average duration of deterministic patterns in the anterior-posterior postural sway (MNL_AP_), *ρ*(21) = .44, *p *= .049. This means that more dynamically stable postural sway occurred when looking at more subjectively beautiful paintings. Ratings of Moved-By are significantly correlated with the average duration of laminar states in the anterior-posterior postural sway (TT_AP_), *ρ*(21) = .45, *p *= .040. This means that longer average intermittent periods in the postural sway, that is, longer periods when postural control gets stuck in a specific state, occurred when looking at more subjectively moving paintings.

### Across-Person Predispositions of Art Experience

In line with our third general expectation, emotional states of being moved and aesthetic appraisals were associated with the COP measures (averaged over all paintings for each of the participants). Specifically, participants who rated paintings higher on Quality, Moved-By, and Drawn-Towards, leaned towards the paintings more (AV_AP_), *ρ*(46) = –0.30, *p *= .045, *ρ*(46) = –0.34, *p *= .022, and *ρ*(46) = –0.30, *p *= .043, respectively. Those who were more emotionally moved by the paintings also had more dynamically stable and complex anterior-posterior sway patterns, *ρ*(46) = 0.31, *p *= .034 (MNL_AP_) and *ρ*(46) = 0.31, *p *= .037 (ENT_AP_). Finally, participants who were more emotionally drawn towards the paintings also had more deterministic, dynamically stable, complex and intermittent anterior-posterior sway patterns, *ρ*(46) = 0.35, *p *= .017 (DET_AP_), *ρ*(46) = 0.40, *p *= .006 (MNL_AP_), *ρ*(46) = 0.41, *p *= .005 (ENT_AP_), and *ρ*(46) = 0.34, *p *= .023 (TT_AP_), respectively.

## Discussion

Postural dynamics did not vary detectably across the entire selection of abstract paintings. However, comparing high-motion (dynamic) and low-motion (static) paintings and zooming in on abstract painting styles exposed differences in several of the postural-sway measures. Arguably the most intriguing overall finding of the present study is that postural dynamics (as a form of embodied engagement with art) correlated in several ways with the measures of the emotional state of being moved and aesthetic appraisal. Systematic *across-painting* and *across-participant* associations were revealed between participants’ emotional states and subjective evaluations of the paintings and their postural control while looking at these paintings. Generally speaking, this implies that the bodily moving and emotionally moving extent of art experiences are associated, as one would expect from an embodied perspective.

The across-painting propensities and across-person predispositions indicate the link between postures and movements in people (i.e., leaning and postural dynamics) and their emotional states related to such postures and movements. They might be providing a first glimpse of what happens during “aesthetic presence” (Menninghaus et al., 2019), considered from an embodied perspective. It is likely that aesthetic presence was stronger or occurred more often for the more beautiful and emotionally moving paintings in this experiment. Accordingly, the correlations suggest that aesthetic presence is associated with a longer average duration of deterministic and intermittent patterns in the dynamics underlying embodied art experiences. Although speculative since the nature and origin of these dynamics have yet to be determined, this might extend or specify the “stopping for knowledge hypothesis” (Sarasso et al., 2020). Longer determinism and intermittency indicate that a system's dynamics becomes more structured for longer periods of time. That this occurs during the attunement with an artwork, hints at a more dynamic and embodied kind of “motor inhibition” related to aesthetic presence. Clearly, this speculation warrants further investigation. Also, what exactly the differences between the across-painting propensities and across-person predispositions are telling us about embodied art experiences is another important line of future research.

An obvious limitation of the present study is the number of statistical tests that were performed. In accordance with the exploratory nature of the study, we decided against making corrections for multiple testing but described all effects with *p *< .05 while at the same time restraining ourselves from strong conclusions. This approach is also in line with one of the main aims of this study, which is to showcase the use of postural sway and this type of methodology in the research on (embodied) art experiences. Nonetheless, it needs to be mentioned that when strong criteria with respect to the correction for multiple statistical testing (e.g., Bonferroni) would be applied, almost all statistical tests for differences between means as well as almost all tests for associations between variables would yield nonsignificant results.

The main contribution of the present study, which extends our previous work (Cox & Van Klaveren, 2024), is the comparison of people's postural dynamics while looking at the most dynamic and most static paintings in the sample, and while looking at three distinct abstract painting styles, labelled fragmentary-abstract, organic-abstract, and dynamic-abstract We hope that this methodology of distinguishing (abstract) paintings styles based on art historical, art theoretical, and/or perceptual criteria in combination with dynamic measurements of body motion as well as self-report of the experience will inspire new research. A possible follow-up study could be targeted at manipulating the potential “movement eliciting” features in paintings and in painting styles. By doing this the extent to which this actually alters people's movement patterns can be investigated. More interestingly though, this will add to our understanding of the nature of the relation between body movement and emotional states of being moved and aesthetic appraisal, as two intertwined aspects of art experiences.

Overall, the present findings add to the empirical evidence in favour of an embodied perspective on art experiences (cf. Brinck, 2018; Burnett & Gallagher, 2020) and how this relates to art-elicited emotional states and aesthetic appraisal. These findings also add to the growing body of research, not only limited to the field of art experience, that people's emotions both influence and are influenced by what happens in or to their body, including bodily movements (cf. Fuchs & Koch, 2014; Schino et al., 2024). More generally, this study demonstrates an empirical approach to studying embodied art experiences, with potential applications in other subfields of perceptual science. It shows that postural dynamics is a spontaneously occurring and easily accessible form of bodily movement which can provide novel insights about art experiences and perceptual phenomena. Finally, techniques for analysing the dynamical organization of behaviour, for instance, RQA as was used here, are a valuable addition to the toolbox of any empirical research field.

## Supplemental Material

sj-docx-1-pec-10.1177_03010066251329918 - Supplemental material for The embodied experience of abstract art: Moving across the 20th century Supplemental material, sj-docx-1-pec-10.1177_03010066251329918 for The embodied experience of abstract art: Moving across the 20th century by Ralf F. A. Cox and Lisa-Maria van Klaveren in Perception

sj-docx-2-pec-10.1177_03010066251329918 - Supplemental material for The embodied experience of abstract art: Moving across the 20th century Supplemental material, sj-docx-2-pec-10.1177_03010066251329918 for The embodied experience of abstract art: Moving across the 20th century by Ralf F. A. Cox and Lisa-Maria van Klaveren in Perception

sj-docx-3-pec-10.1177_03010066251329918 - Supplemental material for The embodied experience of abstract art: Moving across the 20th century Supplemental material, sj-docx-3-pec-10.1177_03010066251329918 for The embodied experience of abstract art: Moving across the 20th century by Ralf F. A. Cox and Lisa-Maria van Klaveren in Perception

## References

[bibr1-03010066251329918] Armstrong R. A. (2014). When to use the Bonferroni correction. Ophthalmic and Physiological Optics, 34(5), 502–508. 10.1111/opo.1213 24697967

[bibr2-03010066251329918] Bertamini M. Byrne C. Bennett K. M. (2013). Attractiveness is influenced by the relationship between postures of the viewer and the viewed person. i-Perception, 4(3), 170–179. 10.1068/i0578 . 23799194 PMC3690408

[bibr3-03010066251329918] Brettell R. R. (1999). Modern Art: 1851–1929. Oxford History of Art.

[bibr4-03010066251329918] Brinck I. (2018). Empathy, engagement, entrainment: The interaction dynamics of aesthetic experience. Cognitive Process, 19(2), 201–213. 10.1007/s10339-017-0805-x PMC5976699 28391411

[bibr5-03010066251329918] Britt D. (2013). Theories of Modern Art: A Source Book by Artists and Critics. University of California Press.

[bibr6-03010066251329918] Burnett M. Gallagher S. (2020). 4E Cognition and the spectrum of aesthetic experience. Journal for the Philosophy of Language, Mind and the Arts, 1(2), 157–176. 10.30687/jolma/2723-9640/2020/02/001

[bibr7-03010066251329918] Clark R. A. Mentiplay B. F. Pua Y. Bower K. J. (2018). Reliability and validity of the Wii Balance Board for assessment of standing balance: A systematic review. Gait & Posture, 61, 40–54. 10.1016/j.gaitpost.2017.12.022 29304510

[bibr8-03010066251329918] Cox R. F. A. Tschacher W. Van Geert P. (2023). Modelling art experiences based on the theory of complex dynamical systems. 10.31219/osf.io/ys4cb

[bibr9-03010066251329918] Cox R. F. A. Van Klaveren L. M. (2024). The embodied experience of abstract art: An exploratory study. Ecological Psychology, 36(2), 111–122. 10.1080/10407413.2024.2355901

[bibr10-03010066251329918] Dewey J. (1934). Art as experience. Minton, Balch.

[bibr11-03010066251329918] Freedberg D. Gallese V. (2007). Motion, emotion and empathy in esthetic experience. Trends in Cognitive Sciences, 11(5), 197–203. 10.1016/j.tics.2007.02.003 17347026

[bibr12-03010066251329918] Fuchs T. Koch S. C. (2014). Embodied affectivity: On moving and being moved. Frontiers in Psychology, 5: 508. 10.3389/fpsyg.2014.00508 24936191 PMC4047516

[bibr13-03010066251329918] Ganczarek J. Pietras K. Cox R. F. A. Ruta N. (2024). Balancing art: The effect of paintings’ conceptual ambiguities on postural control. Psychology of Aesthetics, Creativity, and the Arts. Advance online publication. 10.1037/aca0000717

[bibr14-03010066251329918] Ganczarek J. Ruggieri V. Nardi D. Belardinelli M. O. (2015). Intersection of reality and fiction in art perception: Pictorial space, body sway and mental imagery. Cognitive Processing, 16(1), 233–236. 10.1007/s10339-015-0702-0 26233529 PMC4553148

[bibr15-03010066251329918] Heft H. (2022). Visual art history and the psychology of perception: Perspectivism and its 20th century abandonment in the visual arts and in Gibson’s ecological psychology. Journal of the History of the Behavioral Sciences, 58, 59–84. 10.1002/jhbs.22115 34260748

[bibr16-03010066251329918] Humphries S. Rick J. Weintraub D. Chatterjee A. (2021). Movement in aesthetic experiences: What we can learn from Parkinson disease. Journal of Cognitive Neuroscience, 33(7), 1329–1342. 10.1162/jocn_a_01718 34496397 PMC8925865

[bibr17-03010066251329918] Kapoula Z. Adenis M. Lê T. Yang Q. Lipede G. (2011). Pictorial depth increases body sway. Psychology of Aesthetics, Creativity, and the Arts, 5(2), 186–193. 10.1037/a0022087 .

[bibr18-03010066251329918] Kapoula Z. Gaertner C. (2015). Motion and lateral organization of Monet’s paintings impact body sway. Art and Perception, 3(1), 67–80. 10.1163/22134913-00002014

[bibr19-03010066251329918] Kapoula Z. Lang A. Vernet M. Locher P. (2015). Eye movement instructions modulate motion illusion and body sway with Op Art. Frontiers in Human Neuroscience, 9: 121. 10.3389/fnhum.2015.00121 25859197 PMC4374464

[bibr20-03010066251329918] Koslucher F. Wade M. G. Nelson B. Lim K. Chen F.-C. Stoffregen T. A. (2012). Nintendo Wii Balance Board is sensitive to effects of visual tasks on standing sway in healthy elderly adults. Gait & Posture, 36(3), 605–608. 10.1016/j.gaitpost.2012.05.027 22748469

[bibr21-03010066251329918] Kühnapfel C. Fingerhut J. Brinkmann H. Ganster V. Tanaka T. Specker E. Mikuni J. Güldenpfennig F. Gartus A. Rosenberg R. Pelowski M. (2024). How do we move in front of art? How does this relate to art experience? Linking movement, eye tracking, emotion, and evaluations in a gallery-like setting. Empirical Studies of the Arts, 42(1), 86–146. 10.1177/02762374231160000

[bibr22-03010066251329918] Leder H. Bär S. Topolinski S. (2012). Covert painting simulations influence aesthetic appreciation of artworks. Psychological Science, 23(12), 1479–1481. 10.1177/0956797612452866 23137968

[bibr23-03010066251329918] Lelard T. Stins J. Mouras H. (2019). Postural responses to emotional visual stimuli. Neurophysiologie Clinique / Clinical Neurophysiology, 49(2), 109–114. 10.1016/j.neucli.2019.01.005 30711434

[bibr24-03010066251329918] Menninghaus W. Wagner V. Wassiliwizky E. Schindler I. Hanich J. Jacobsen T. Koelsch S. (2019). What are aesthetic emotions? Psychological Review, 126(2), 171–195. 10.1037/rev0000135 30802122

[bibr25-03010066251329918] Merleau-Ponty M. (2010). Phenomenology of perception. (D. Landes, Trans.; 1st ed.). Routledge. 10.4324/9780203720714

[bibr26-03010066251329918] Pavan P. Cardaioli M. Ferri I. Gobbi E. Carraro A. (2014). A contribution to the validation of the Wii Balance Board for the assessment of standing balance. European Journal of Sport Science, 15(7), 600–605. 10.1080/17461391.2014.956801 25222558

[bibr27-03010066251329918] Pellecchia G. L. (2003). Postural sway increases with attentional demands of concurrent cognitive task. Gait & Posture, 18(1), 29–34. 10.1016/S0966-6362(02)00138-8 12855298

[bibr28-03010066251329918] Ramenzoni V. C. Riley M. A. Shockley K. Chiu C.-Y. P. (2007). Postural responses to specific types of working memory tasks. Gait & Posture, 25(3), 368–373. 10.1016/j.gaitpost.2006.04.014 16806935

[bibr29-03010066251329918] Rosenbaum D. Mama Y. Algom D. (2017). Stand by your Stroop: Standing up enhances selective attention and cognitive control. Psychological Science, 28(12), 1864–1867. 10.1177/0956797617721270 28952883

[bibr30-03010066251329918] Salihu A. T. Hill K. D. Jaberzadeh S. (2022). Effect of cognitive task complexity on dual task postural stability: A systematic review and meta-analysis. Experimental Brain Research, 240(3), 703–731. 10.1007/s00221-021-06299-y 35034175

[bibr31-03010066251329918] Sarasso P. Neppi-Modona M. Sacco K. Ronga I. (2020). Stopping for knowledge”: The sense of beauty in the perception-action cycle. Neuroscience & Biobehavioral Reviews, 118, 723–738. 10.1016/j.neubiorev.2020.09.004 32926914

[bibr32-03010066251329918] Schino G. Van Klaveren L. Gallegos González H. Cox R. F. A. (2024). Applying bodily sensation maps to art-elicited emotions: An explorative study. Psychology of Aesthetics, Creativity, and the Arts, 18(3), 315–329. 10.1037/aca0000444

[bibr33-03010066251329918] Van Geert E. Wagemans J. (2020). Order, complexity, and aesthetic appreciation. Psychology of Aesthetics, Creativity, and the Arts, 14(2), 135–154. 10.1037/aca0000224

[bibr34-03010066251329918] Wölfflin H. (2015). Principles of Art History: The Problem of the Development of Style in Early Modern Art. Trans Jonathan Blower 100th Anniversary Edition. Getty Research Institute.

