# Supplementary material for: The embodied experience of abstract art: Moving across the 20th century
Source: Perception. 2025 Apr 3;54(6):431–40. doi: 10.1177/03010066251329918 (PMC12096175; doi:10.1177/03010066251329918)
Supplement: sj-docx-1-pec-10.1177_03010066251329918 - Supplemental material for The embodied experience of abstract art: Moving across the 20th century [file sj-docx-1-pec-10.1177_03010066251329918.docx]

**Supplement 1**

**Overview of the artworks**

| 1 | Vasily Kandinsky  Violet-Orange  1935  Oil on canvas  88.9 cm x 116.2 cm | 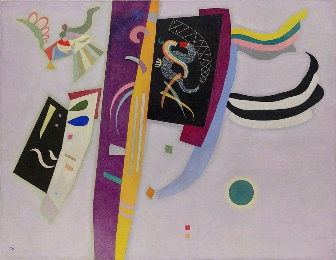 |
| --- | --- | --- |
| 2 | Alberto Magnelli  Lyric Explosion No. 14: Intoxicated Man  1918  Oil on canvas  110.5 cm x 135.3 cm | 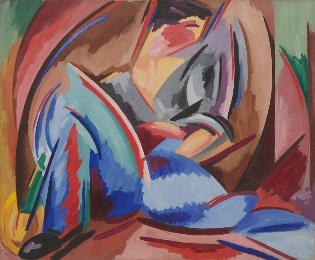 |
| 3 | László Moholy-Nagy  K VII  1922  Oil paint and graphite on canvas  115.3 cm x 135.9 cm | 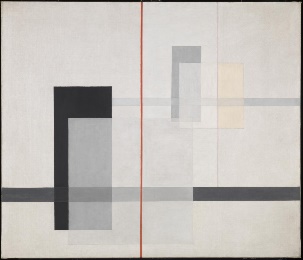 |
| 4 | Fahrelnissa Zeid  Untitled  c. 1950s  Oil paint on canvas  182 cm x 222 cm | 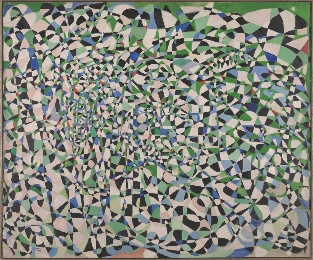 |
| 5 | Willem de Kooning  … Whose Name Was Writ in Water  1975  oil paint on canvas  195 cm x 223 cm | 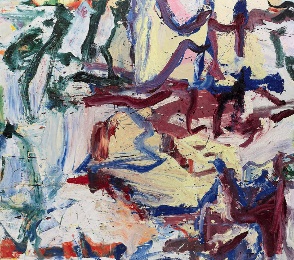 |
| 6 | Lee Ufan  From Line  1978  Oil paint and glue on canvas  181.8 cm x 227.5 cm | 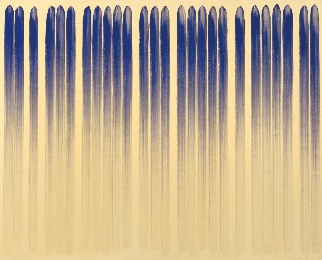 |
| 7 | Ben Nicholson  June 1937  1937  Oil paint on canvas  159.4 cm x 201.3 cm | 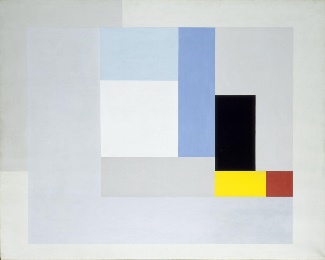 |
| 8 | John Piper  Abstract I  1935  Oil paint on canvas on plywood  91.7 cm x 106.5 cm | 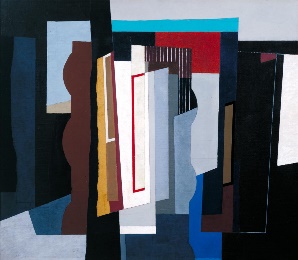 |
| 9 | Piet Mondriaan  Compositie No.II  1913  oil paint on canvas  85 cm x 115 cm | 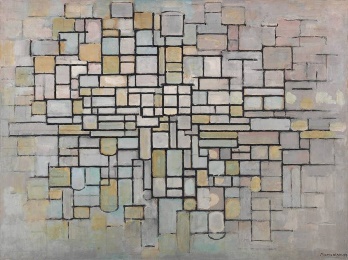 |
| 10 | Gerhard Richter  St John  1988  oil paint on canvas  201 cm x 261 cm | 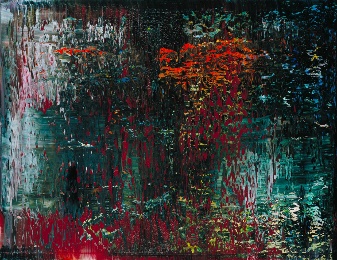 |
| 11 | Karel Appel  Amorous Dance  1955  oil paint on canvas  114 cm x 146 cm | 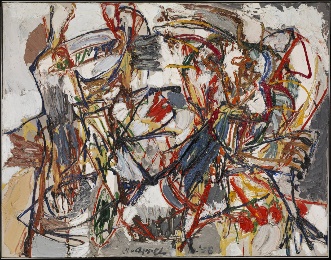 |
| 12 | Jean-Paul Riopelle  Perspectives  1956  oil paint on canvas  80 cm x 100 cm | 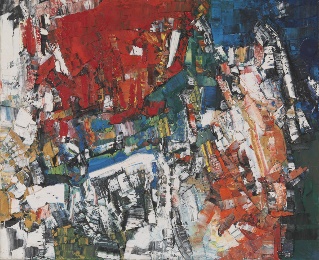 |
| 13 | Bryan Wynter  Meander  1967  oil paint on canvas  168 cm x 213 cm | 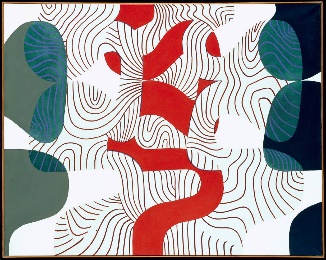 |
| 14 | Jackson Pollock  Yellow Islands  1952  Oil paint on canvas  143.5 cm x 185.4 cm | 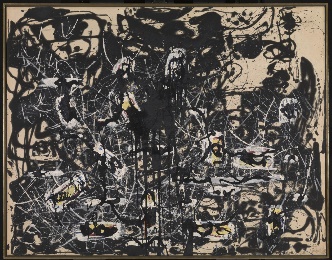 |
| 15 | Robert Delaunay  Window on the City No. 3  1911/12  oil paint on canvas  114 cm x 131 cm | 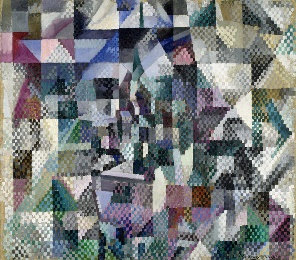 |
| 16 | Lyubov Popova  Painterly Architectonic  1917  Oil on canvas  80 cm x 98 cm | 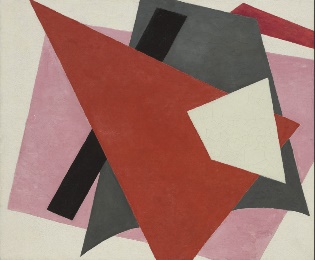 |
| 17 | Howard Hodgkin  Dinner at West Hill  1964–6  Oil paint on canvas  106.7 cm x 127 cm | 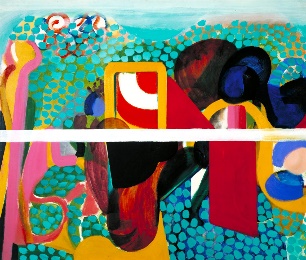 |
| 18 | Cecily Brown  Trouble in Paradise  1999  Oil paint on canvas  191.2 cm x 229.3 cm | 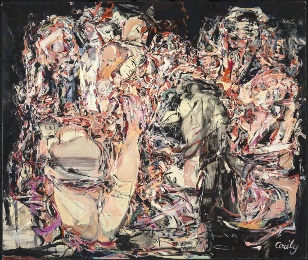 |
| 19 | Bridget Riley  Nataraja  1993  Oil paint on canvas  165.1 cm x 227.7 cm | 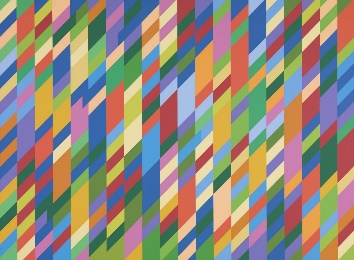 |
| 20 | Lee Krasner  Gothic Landscape  1961  Oil paint on canvas  176.8 cm x 237.8 cm | 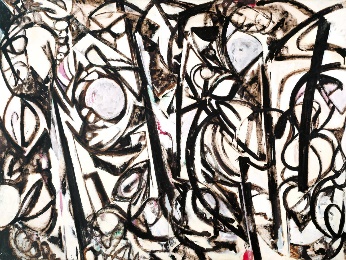 |
| 21 | Adolph Gottlieb  Labyrinth No. 2  1950  Oil paint on canvas  91.4 cm x 121.9 cm | 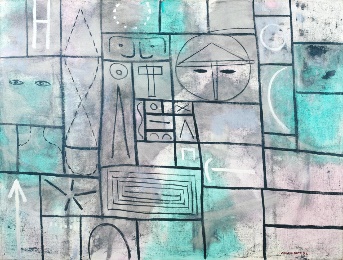 |
