# Supplementary material for: The embodied experience of abstract art: Moving across the 20th century
Source: Perception. 2025 Apr 3;54(6):431–40. doi: 10.1177/03010066251329918 (PMC12096175; doi:10.1177/03010066251329918)
Supplement: sj-docx-2-pec-10.1177_03010066251329918 - Supplemental material for The embodied experience of abstract art: Moving across the 20th century [file sj-docx-2-pec-10.1177_03010066251329918.docx]

**Supplement 2**

**Recurrence Quantification Analysis**

Recurrence quantification analysis is a nonlinear time-series technique which combines two subsequent analysis steps. First, attractor reconstruction, using a delay-embedding technique applied on a continuous data stream (Takens, 1981). Second, recurrence quantification, which analyses the recurrent structure as displayed in a recurrence plot (RP; Eckmann et al., 1987). RPs are created by tracking all the recurrent states of the reconstructed attractor. Recurrent states are areas in the phase space that the systems revisits at different times during a period of observation. In this way, RQA results in a set of measures that inform about different aspects of the dynamic organisation of a system. A number of these measures will be discussed below. Before RQA can be executed, a number of parameters need to be set, viz. the delay and embedding dimension for the attractor reconstructions, and the radius or fixed recurrence rate and minimum line length for the recurrence quantification.

In this study we used a delay of 70 and an embedding dimension of 6, which were selected on the basis of the Average Mutual Information (Fraser & Swinney, 1986) and False Nearest Neighbor (Kennel et al., 1992) procedures, respectively. Furthermore, we used a variable radius in phase space, which provided recurrence rates of 0.10 for each trial (cf. Riley et al., 1999; Wijnants et al., 2009). Finally, the minimum length of the diagonal and vertical lines in the RP was set to 20, so as to avoid relatively short and random patterns which lead to saturation in the line measures (Almeida et al., 2018, Thiel et al., 2002; Tommasini et al., 2022). This means that our analysis was based on the identification of recurrent patterns (i.e. lines) of at least 200 ms long. Recurrent patterns indicate deterministic episodes of various duration in the dynamics of the underlying system, which appear as diagonal and vertical lines in the RP.

The next step in the analysis is to quantify the recurrent patterns in the RP. In this study we considered five recurrence measures, for each COP movement direction: Determinism, Meanline, Entropy, Laminarity, and Trapping Time. Determinism (DET_ML_ and DET_AP_) is defined as the ratio of the number of recurrent points forming recurrent patterns on diagonal lines (of minimally 20 recurrent points) over the total number of recurrent points. This measure informs about the extent of deterministic behaviour of the system. Meanline (MNL_ML_ and MNL_AP_) is the average duration of diagonal recurrent patterns during the measurement, that is, the average length of diagonal lines. It is considered a measure of dynamic stability of the system. Entropy (ENT_ML_ and ENT_AP_) is computed as the Shannon entropy of the distribution of line lengths of the diagonal lines in the RP (cf. Webber & Zbilut, 1994). Entropy quantifies the dynamic complexity of the system. Laminarity (LAM_ML_ and LAM_AP_) and Trapping Time (TT_ML_ and TT_AP_) capture the proportion and average duration of laminar states (of minimally 200 ms), respectively. Laminar states represent intermittency or rigidity in the system, that is, when the system gets stuck in one or more states for some time. Laminarity is calculated similarly as Determinism except that it reflects the proportion of recurrent points on vertical lines in the RP. Trapping Time is similar to Meanline but quantifies the average vertical line length, and therefore the average duration of vertical recurrent patterns during the measurement. More detailed descriptions of recurrence quantification analysis including descriptions of all measures can be found elsewhere (e.g. Arellano-Véliz et al., 2024; Marwan et al., 2007; Riley et al., 1999; Wallot & Leonardi, 2018; Webber & Zbilut, 2005; Wijnants et al., 2012). The recurrence quantification analysis was performed using custom-made Matlab scripts in combination with a function from Marwan’s CRP Toolbox (<http://www.recurrence-plot.tk>).

References

Arellano-Véliz, N. A., Cox, R. F. A., Jeronimus, B. F., Castillo, R. D., & Kunnen, E. S. (2024). Personality expression in body motion dynamics: An enactive, embodied and complex systems perspective. *Journal of Research in Personality, 110*: 104495. <https://doi.org/10.1016/j.jrp.2024.104495>

Almeida, T. P., Schlindwein, F. S., Salinet, J., Li, X., Chu, G. S., Tuan, J. H., Stafford, P. J., Ng, G. A., & Soriano, D. C. (2018). Characterization of human persistent atrial fibrillation electrograms using recurrence quantification analysis. *Chaos Interdisciplinary Journal Nonlinear Science, 28(8)*: 085710. <https://doi.org/10.1063/1.5024248>

Eckmann, J. P., Kamphorst, S. O., & Ruelle, D. (1987). Recurrence plots of dynamical systems. *Europhysics Letters, 5*, 973–977. doi:10.1209/0295-5075/4/9/004

Fraser, A. M., & Swinney, H. L. (1986). Independent coordinates for strange attractors from mutual information. *Physical Review A, 33*, 1134–1140. doi:10.1103/PhysRevA.33.1134

Kennel, M. B., Brown, R., & Abarbanel, H. D. I. (1992). Determining embedding dimension for phase-space reconstruction using a geometrical construction. *Physical Review A, 45*, 3403–3411. doi:10.1103/PhysRevA.45.3403

Marwan, N., Romano, M. C., Thiel, M., & Kurths, J. (2007). Recurrence in complex systems. *Physics Reports, 438*, 237–329. doi:10.1016/j.physrep.2006.11.001

Riley, M. A., Balasubramaniam, R., & Turvey, M. T. (1999). Recurrence quantification analysis of postural fluctuations. *Gait & Posture, 11*, 12–24. doi:10.1016/S0966-6362(98)00044-7

Takens, F. (1981). Detecting strange attractors in fluid turbulence. In D. A. Rand & L. S. Young (Eds.), Dynamic Systems and Turbulence (pp. 366–381). New York: Springer. doi:10.1007/BFb0091924

Thiel, M., Romano, M. C., Kurths, J., Meucci, R., Allaria, E., & Arecchi, F. T. (2002). Influence of observational noise on the recurrence quantification analysis. *Physica D, 171(3)*, 138–152.

Tommasini, F. C., Evin, D. A., Bermejo, F., Hüg, M. X., Barrios, M. V., & Pampaluna, A. (2022). Recurrence analysis of sensorimotor trajectories in a minimalist perceptual task using sonification. *Cognitive Processing.* https://doi.org/10.1007/s10339-021-01068-9

Wallot, S., & Leonardi, G. (2018). Analyzing multivariate dynamics using cross-recurrence quantification analysis (CRQA), diagonal-crossrecurrence profiles (DCRP), and multidimensional recurrence quantification analysis (MDRQA)–a tutorial in R. *Frontiers in Psychology, 9*: 2232. <https://doi.org/10.3389/fpsyg.2018.02232>

Webber Jr, C. L., & Zbilut, J. P. (1994). Dynamical assessment of physiological systems and states using recurrence plot strategies. *Journal of Applied Physiology, 76*, 965–973. <https://doi.org/10.1152/jappl.1994.76.2.965>

Webber Jr, C. L., & Zbilut, J. P. (2005). Recurrence quantification analysis of nonlinear dynamical systems. In M. A. Riley and G. C. Van Orden (Eds.), Tutorials in contemporary nonlinear methods for the behavioral sciences (pp. 142–177). Alexandria, VA: NSF

Wijnants, M.L., Bosman, A.M.T., Hasselman, F., Cox, R.F.A., & Van Orden, G.C. (2009). 1/f scaling fluctuation in movement time: Temporal structure changes with massed practice. *Nonlinear Dynamics, Psychology, and Life Sciences, 13(1)*, 79-98.

Wijnants, M.L., Hasselman, F., Cox, R.F.A., Bosman, A.M.T., & Van Orden, G.C. (2012). An interaction-dominant perspective on reading fluency and dyslexia. *Annals of Dyslexia, 62(2)*, 100-119.
