# Supplementary material for: The embodied experience of abstract art: Moving across the 20th century
Source: Perception. 2025 Apr 3;54(6):431–40. doi: 10.1177/03010066251329918 (PMC12096175; doi:10.1177/03010066251329918)
Supplement: sj-docx-3-pec-10.1177_03010066251329918 - Supplemental material for The embodied experience of abstract art: Moving across the 20th century [file sj-docx-3-pec-10.1177_03010066251329918.docx]

**Supplement 3**

**Descriptions of the three abstract painting styles**

**Fragmentary-abstract**

**Definition:** Artworks categorized as fragmentary-abstract are characterized by broken, disjointed, or segmented compositions that often employ geometric or angular forms. These works focus on deconstructing reality and disrupting traditional visual unity. It may appear calculated, often relating to urbanization, technology, and mathematics. Creating tension or balance between order and chaos, they may invite the viewer to reconstruct meaning from fragmented elements.

| 4 | Fahrelnissa Zeid - *Untitled (c. 1950s)*   - **Painting Style**: Zeid’s work employs fragmented geometric forms and intricate patterns, reflecting her engagement with both Western abstraction and Islamic art traditions.. - **Historical Context**: Zeid’s work bridges Eastern and Western artistic practices, symbolizing her personal identity as a Turkish artist working in Europe. - **Artist’s Intentions**: Zeid intended to explore the interplay between structure and chaos, often reflecting a universal quest for harmony amidst fragmentation. | 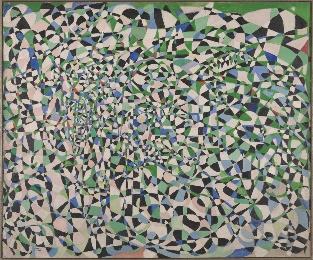 |
| --- | --- | --- |
| 9 | Piet Mondriaan - *Compositie No.II (1913)*   - **Painting Style**: This early Mondrian work employs fragmented, overlapping planes that suggest three-dimensionality while remaining abstract. - **Historical Context**: Created during Mondrian’s transition from figurative painting to abstraction, this work reflects his exploration of Cubism geometries. - **Artist’s Intentions**: Mondrian intended to distil visual reality into its essential structures, aiming for a universal artistic language. | 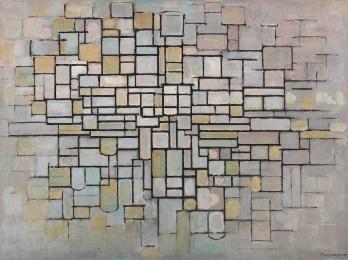 |
| 15 | Robert Delaunay - *Window on the City No. 3 (1911/12)*   - **Painting Style**: Delaunay employs fractured, colourful shapes to depict an urban scene, merging Cubism and Orphism to create dynamic energy. - **Historical Context**: Created during the rise of modernism, the work reflects Delaunay’s fascination with the changing urban environment and its visual rhythm. - **Artist’s Intentions**: Delaunay intended to capture the vibrancy and fragmented reality of modern cities. | 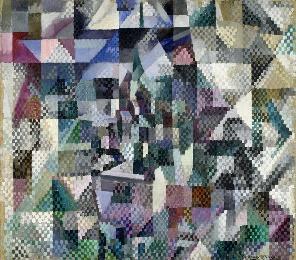 |
| 19 | Bridget Riley - *Nataraja (1993)*   - **Painting Style**: Riley’s optical abstraction employs overlapping wave patterns, creating a fragmented visual experience. - **Historical Context**: Created during a period of experimentation with perceptual effects, Riley’s work draws on Op-art’s focus on visual perception and movement. - **Artist’s Intentions**: Riley intended to challenge the viewer’s perception through carefully calculated visual fragmentation. | 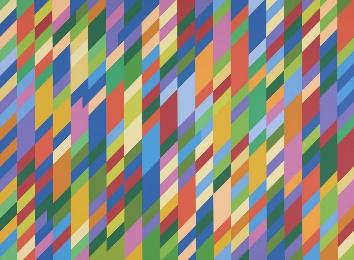 |

**Organic-abstract**

**Definition:** Artworks categorized as organic-abstract artworks are characterized by fluid, biomorphic, and natural forms, mimicking patterns and rhythms found in nature. These compositions suggest movement, growth, and transformation, evoking harmony and interconnectedness through flowing structures. It may appear less calculated and more organic, often relating to natural processes and/or flow (i.e., in music). By blurring boundaries between abstraction and nature, they may invite an immersive experience that resonates with the viewer on a physical and emotional level.

| 1 | Vasily Kandinsky - *Violet-Orange (1935)*   - **Painting Style**: Kandinsky’s work employs biomorphic shapes and fluid compositions, reflecting his interest in synesthesia and spiritual abstraction. - **Historical Context**: Created during his Bauhaus period, this work represents a shift towards playful, organic forms in contrast to earlier geometric abstractions. - **Artist’s Intentions**: Kandinsky intended to evoke emotional resonance through abstract forms and colors. | 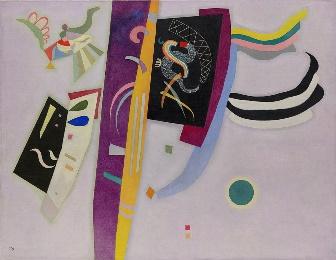 |
| --- | --- | --- |
| 2 | Alberto Magnelli - *Lyric Explosion No. 14: Intoxicated Man (1918)*   - **Painting Style**: Magnelli employs abstracted organic forms and rhythmic composition, influenced by Futurism and early abstraction. - **Historical Context**: Created post-WWI, this work reflects a desire to move away from realism towards expressive abstraction. - **Artist’s Intentions**: Magnelli intended to convey emotional intensity and lyricism through abstraction. | 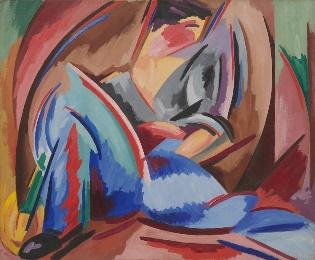 |
| 13 | Bryan Wynter - *Meander (1967)*   - **Painting Style**: Wynter employs abstract, flowing lines and shapes to evoke natural landscapes and water currents. - **Historical Context**: Created during a time when abstraction was becoming more introspective, Wynter’s work reflects an interest in natural processes. - **Artist’s Intentions**: Wynter intended to connect the viewer with the rhythms and patterns of nature. | 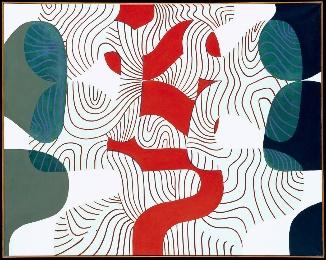 |
| 18 | Cecily Brown - *Trouble in Paradise (1999)*   - **Painting Style**: Brown’s painterly abstraction employs sensual, organic forms with references to the human body and natural landscapes. - **Historical Context**: Emerging at the turn of the millennium, this work reflects a renewed interest in expressive, gestural abstraction. - **Artist’s Intentions**: Brown intended to blur the boundaries between abstraction and figuration, creating evocative and tactile experiences. | 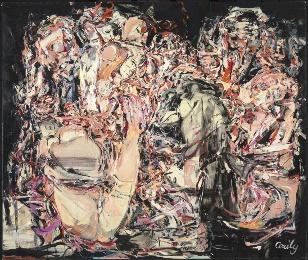 |

**Dynamic-abstract**

**Definition:** Artworks categorized as dynamic-abstract artworks are characterized by their sense of motion, energy, and visual rhythm, often achieved through vigorous gestures, overlapping layers, and spontaneous compositions. It may appear as letting go of concrete references, often focussing on the process, chance and energy. These works capture the vitality and flux of transformation that may draw the viewer into an energetic and immersive experience.

| 5 | Willem de Kooning - *… Whose Name Was Writ in Water (1975)*   - **Painting Style**: De Kooning employs gestural brushstrokes and dynamic layering to create a sense of movement and flux. - **Historical Context**: Created during the height of Abstract Expressionism, reflecting a fascination with motion and spontaneity. - **Artist’s Intentions**: De Kooning aimed to capture the essence of transience and impermanence. | 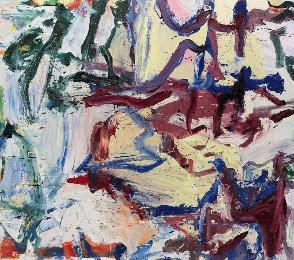 |
| --- | --- | --- |
| 11 | Karel Appel - *Amorous Dance (1955)*   - **Painting Style**: Appel employs bold, gestural brushstrokes, vibrant colour contrasts, and a sense of raw energy. The composition has a spontaneous and rhythmic quality, evoking movement and vitality. - **Historical Context**: Created during the height of the Cobra-movement, Appel’s work reflects the group’s emphasis on spontaneity, emotion, and a childlike, untamed aesthetic. **Artist’s Intentions**: Appel intended to capture the primal and expressive nature of human emotion, using abstraction as a means to convey unfiltered vitality and freedom. | 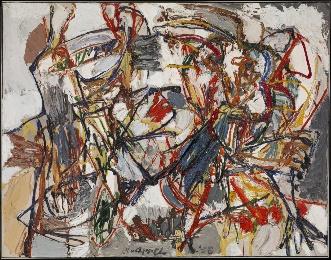 |
| 12 | Jean-Paul Riopelle - *Perspectives (1956)*   - **Painting Style**: Riopelle employs dense layers of paint applied with a palette knife, creating a dynamic, textured surface. The composition pulses with energy and movement, with overlapping colours and forms. - **Historical Context**: Riopelle sought to push the boundaries of gestural abstraction during the post-WWII era - **Artist’s Intentions**: Riopelle intended to convey a sense of infinite depth and motion, using abstraction to explore emotional and spatial dimensions. | 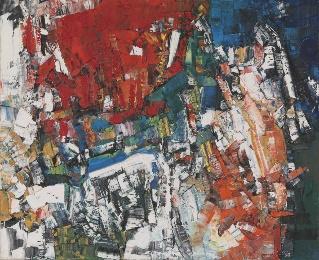 |
| 14 | Jackson Pollock - *Yellow Islands (1952)*   - **Painting Style**: Pollock employs a drip painting technique, featuring layered, dynamic networks of paint with areas of vibrant yellow breaking through the energetic composition. **Historical Context**: Created during Pollock’s mature period, this work embodies his approach to action painting, where physical gestures became integral to the creative process. - **Artist’s Intentions**: Pollock intended to express subconscious impulses and the raw energy of existence through his gestural abstraction. | 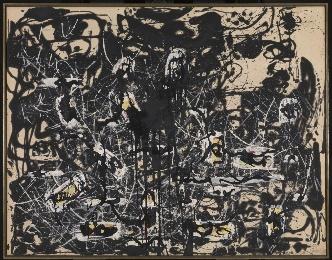 |
| 20 | Lee Krasner - *Gothic Landscape (1961)*   - **Painting Style**: Krasner employs bold, gestural strokes and fragmented organic shapes, creating a composition that feels both turbulent and structured. - **Historical Context**: Created after the death of her husband, Jackson Pollock, this work reflects Krasner’s exploration of grief and renewal. - **Artist’s Intentions**: Krasner intended to channel intense emotions and capture the dynamism of internal and external landscapes, emphasizing resilience and transformation. | 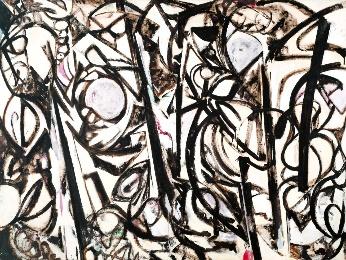 |
